# Metacognitive deficits in major depressive disorder

**DOI:** 10.3389/fpsyt.2025.1524046

**Published:** 2025-03-14

**Authors:** Shuning Hong, Mengjiao Chi, Haisi Chen, Fengfeng Chu, Yuping Zheng, Ming Tao

**Affiliations:** ^1^ Second Clinical Medical School, Zhejiang Chinese Medical University, Hangzhou, China; ^2^ Affiliated Mental Health Center & Hangzhou Seventh People’s Hospital, Zhejiang University School of Medicine, Hangzhou, China; ^3^ Department of Sleep Disorders, The Fifth People's Hospital of Lin'an District, Hangzhou, China

**Keywords:** metacognition, metacognitive ability, major depressive disorder, neuropsychology, mental rotation

## Abstract

**Objective:**

We aimed to investigate the metacognition of patients with major depressive disorder (MDD) and its correlation with their condition, as well as explore its diagnostic significance in the early stages of the disease, thereby providing a reference for clinical treatment.

**Methods:**

Using a cross-sectional research design, we selected 66 patients diagnosed with MDD and 99 healthy controls for a mental rotation task; we examined their metacognitive performance using a post-decisional confidence assessment paradigm. We evaluated various aspects, including their performance on first-order tasks (d’), metacognitive bias (average confidence), metacognitive sensitivity (meta-d’), metacognitive efficiency (the M Ratio).

**Results:**

In terms of the first-order task performance (d’), the group with MDD scored significantly lower than the healthy controls (t = -4.274, p < 0.001, respectively). Regarding metacognitive bias(average confidence), metacognitive sensitivity (meta-d’), and metacognitive efficiency (the M ratio), the group with MDD performed significantly worse than the healthy controls (t = -4.280, p < 0.001; t = -3.540, p < 0.001; t = -2.104, p = 0.039, respectively). In addition, the Hamilton Rating Scale for Depression (HAMD-17) scores of the patients with MDD were significantly negatively correlated with their d’, average confidence levels, meta-d’, and M ratio(r = -0.468, p < 0.001; r = -0.601, p < 0.001;r = -0.457, p < 0.001; r = -0.362, p = 0.003), The average confidence levels of MDD patients are significantly positively correlated with d’, meta-d’, and M ratio. (r = -0.552, p < 0.001; r = 0.738, p < 0.001;r =0.273, p =0.02).

**Conclusion:**

The metacognitive abilities of patients with MDD were significantly impaired, and the degree of metacognitive impairment was related to the severity of clinical depressive symptoms. Moreover, the impairment of their metacognitive abilities was correlated with negative metacognitive bias.

**Clinical Trial Registration:**

https://www.chictr.org.cn, identifier ChiCTR2400091242

## Introduction

In recent years, human cognition has become a trending topic in research, with much attention paid to the abnormalities in metacognition across a range of mental health conditions. Flavell introduced the concept of “metacognition” in 1979 (1). It was originally described as an individual’s awareness of their own cognitive processes. For instance, in the process of learning, individuals engage in various cognitive activities such as memorizing, decision-making, and conducting operations, while at the same time actively monitoring and regulating these activities. This re-memorizing, re-decision-making, and re-conducting operations of cognitive activities themselves is referred to as metacognition. The strength of metacognitive ability directly affects an individual’s capacity to assess and adjust their own cognitive performance.

Metacognition does not necessarily correlate with cognitive performance. Some people may be very good at a particular cognitive task without realizing that they are performing well, thus lacking confidence in their answers. On the contrary, others may perform poorly on the same task without realizing it. Regardless of how well these people perform on a cognitive task, they have low metacognitive abilities. Meanwhile, individuals with good metacognition do not have to perform cognitive tasks at a high level. Good metacognition is reflected in their ability to realize how well the task is being performed and adjust their confidence levels accordingly (2). These confidence levels can be used to gauge metacognition. In quantifying metacognition, three indicators can be employed: metacognitive sensitivity, metacognitive efficiency, and metacognitive bias (3). These concepts are defined below.

Metacognitive bias is the difference in confidence when performance on a basic task remains consistent. If confidence is assessed following a judgment about decision options A or B, high metacognitive bias will result in an overall high level of confidence for overconfident individuals, regardless of the previous judgment about decision-making. Metacognitive sensitivity can be assessed by the extent to which an observer’s confidence ratings predict their actual success (4). We can consider a person to have high metacognitive sensitivity if their confidence ratings are high after correct judgments and low after incorrect judgments. Metacognitive efficiency is defined as metacognitive sensitivity relative to an individual’s task performance. There is a specific level of metacognitive efficiency in an individual in a particular domain (such as memory and decision-making) and that this efficiency is not correlated with the level of task performance. Therefore, We quantify metacognitive efficiency (M-ratio) as the ratio of metacognitive sensitivity (meta-d’) to first-order task performance (d-prime). Prior research on metacognition has predominantly relied on statistical correlation coefficients, such as gamma correlation and phi correlation, to measure metacognitive ability (5). While these calculations are intuitive, they do not fully distinguish metacognitive sensitivity from metacognitive bias, and these measures are susceptible to the adverse influence of factors such as response bias. Later, Fleming provided an unbiased measure of sensitivity based on Signal Detection Theory(SDT)and Receiver Operating Characteristic (ROC) curve analysis (6). This method enables a more precise distinction between metacognitive bias, sensitivity, and efficiency. In 2017, Fleming developed a hierarchical Bayesian estimation to evaluate metacognitive efficiency (7). This technique incorporates a generative confidence model within the SDT framework to quantify metacognitive sensitivity (meta-d’). It enhances statistical power while addressing the limitation of prior methods that necessitated extensive confidence data to achieve robust parameter estimates.

The metacognitive profile of patients with MDD is fundamentally different from that of the healthy population and persists even after symptoms have resolved (8). Crucially, metacognitive beliefs refer to a second-order system for assessing an individual’s own cognitive processes and mental states, and are characterized by 1) Cognitive regulation beliefs: beliefs about the ability to control thinking (e.g., “I can’t stop worrying”). 2) Cognitive appraisal beliefs: judgments about the dangerousness/helpfulness of particular cognitive contents (e.g. “Repeatedly thinking about failure prevents mistakes”). 3) Cognitive monitoring beliefs: assessments of the effectiveness of self-cognitive monitoring (e.g., ‘I can’t tell if I’m making the right decisions’)-may mediate the disorder. Whereas traditional cognitive theories emphasize dysfunctional attitudes (e.g., perfectionism, excessive need for approval) in depressed individuals (6), contemporary evidence reveals the following distinction: whereas dysfunctional attitudes represent a first-order negative self-schema (“I’m a failure”), metacognitive beliefs constitute a second-order cognitive regulatory appraisal (“Thinking about failure overwhelms me”).A study by Yilmaz et al. (2015) found that while dysfunctional attitudes predicted depressive symptoms, their impact was weaker than metacognitive beliefs (9). This suggests that metacognitive beliefs may account for a greater proportion of the variance in depressive symptoms than dysfunctional attitudes, which have been emphasized by traditional cognitive theories. (This is confirmed by a study by Leach et al. (2019) (10), who found that perinatal depressive symptoms were associated with dysfunctional attitudes specific to the role of motherhood, but that metacognitive beliefs - particularly negative beliefs about the uncontrollability and dangerousness of worry - were more predictive of depressive symptoms, which may be due to impaired self-efficacy calibration (metacognitive bias) and a decreased ability to discern right and wrong decision-making (meta-d’). This further confirms the independent influence of metacognitive beliefs on depressive symptoms.

Researchers have made notable progress in exploring metacognitive biases. However, few studies exist on the effects of depressive emotions on metacognitive sensitivity and efficiency, and the existing literature often lacks depth and comprehensiveness. Some findings suggest that symptom dimensions related to anxiety and depression are associated with low self-confidence and metacognitive efficiency (11, 12). However, a follow-up study by Moses-Payne et al. (13) in 2019 did not find an association between metacognitive sensitivity (meta-d’) and metacognitive efficiency (M Ratio) and depressive symptoms, despite validating that a lower confidence level was associated with more intense depression. And in another study, researchers induced negative emotions related to sadness in healthy participants through methods such as recalling sad events, viewing sad images, and watching sad videos (14). Subsequently, participants were administered a metacognitive task. The results showed that the induction of sadness had no significant effect on task completion. Although sadness significantly reduced participants’ confidence levels, and it did not affect metacognitive efficiency (M Ratio). These negative findings suggest that the relationship between depressive symptoms and metacognitive processes requires further exploration and clarification.

To further investigate the characteristics of metacognitive impairments in patients with depressive disorders and their correlation with clinical symptomatology, our study employed the mental rotation task as the primary cognitive assessment tool. Mental rotation, a term in cognitive psychology, refers to the mental process of imagining the rotation of objects in two-dimensional or three-dimensional space. This task was first introduced by Shepard and Metzler in 1971 and involves presenting pairs of objects rotated at specific angles relative to each other, with participants required to determine whether the two objects are identical or mirror images (15). The cognitive processes underlying this task can be divided into the following stages: creating a mental image, mentally rotating the image; comparing the images, deciding whether the objects are the same, and making a decision (16). This task not only effectively evaluates an individual’s spatial cognitive abilities but also allows for a deeper examination of participants’ metacognitive performance through post-decision confidence assessments, including task performance, metacognitive bias (Average Confidence), metacognitive sensitivity (Meta-d’), and metacognitive efficiency (M Ratio).

We hypothesize that individuals with depression exhibit impaired metacognitive function, and the degree of metacognitive impairment is correlated with the severity of clinical depressive symptoms as well as with negative metacognitive bias(Average Confidence).By employing the mental rotation task, we aim to explore the characteristics of metacognitive dysfunction in patients with major depressive disorder (MDD)and its correlation with the severity of the illness, thereby providing a theoretical foundation for future longitudinal studies on early identification and prognostic assessment.

## Methods

### Participants

We included 66 patients with MDD (HAMD-17 >17 points) and 99 healthy controls (HAMD-17 <7 points), with an equal proportion of men and women; most of the participants were unmarried, except for 2 patients with MDD. The participants were aged 18-60 years. The average age of the group with MDD was 20.68 ± 0.64 years, while that of the healthy control group was 21.24 ± 0.18 years. There were no statistically significant differences between the MDD patients and healthy controls in terms of gender (p=0.799) or age (p=0.759), as shown in **Table 1**. Depression levels were assessed using the Hamilton Depression Rating Scale (HAMD-17). Participants in the MDD group exhibited a mean score of 22.98 ± 0.47, whereas those in the healthy control group had a mean score of 0.87 ± 0.13. The difference between the two groups was highly significant (P < 0.001). The mental rotation task was used to assess the participants’ metacognition.

**Table 1:** Demographic information and HAMD-17 results of the patients with MDD and the healthy controls.

	Group with MDD (n=66)	Healthy controls (n=99)	t	p
Sex (m/f)	32/34	50/49		0.799
Age	20.68 ± 0.64	21.24 ± 0.18	0.662	0.759
HAMD-17	22.98 ± 0.47	0.87 ± 0.13	45.449	<0.001

This study was approved by the Medical Research Ethics Committee of the Seventh People’s Hospital of Hangzhou, all subjects participated in the study voluntarily, and the advantages and disadvantages of the study were analysed for each subject and his/her guardian, and the subjects and their guardians were informed of the purpose of the study, the process of the study and the possible discomforts and risks in the course of the study by the experimental personnel before the experiment. Given that individuals with severe major depressive disorder (MDD) may have impaired capacity to provide informed consent, the study ensured that the autonomy of all participants was fully respected. All participants with severe MDD and their guardians provided written informed consent. Participants in the healthy control group provided written informed consent themselves. The informed consent form informed participants in detail of the experimental procedures and safety measures, and they were free to withdraw from the experiment at any time.

Depression group: derived from patients with depressive disorders who attended the Seventh People’s Hospital of Hangzhou from January 2024 to July 2024, all subjects were diagnosed and included in the study group in accordance with the criteria for inclusion by two senior attending physicians and above. Inclusion criteria: (1) meet the diagnostic criteria for depressive episodes and recurrent depressive disorders in ICD-10; (2) outpatients or inpatients, aged 18-60 years old; (3) have at least junior high school education or above, and are able to cooperate in completing the cognitive function test and clinical assessment; (4) have a total score of 17 or above on the HAMD-17 at the time of enrollment; and (5) have the consent of the patients and their families, and sign an informed consent form. Exclusion Criteria: (1) Pregnant or lactating women; (2) Patients with depressive disorders with psychotic symptoms and suicidal tendency; (3) Patients with serious physical illnesses, such as serious liver and kidney diseases, heart diseases, hypertension, diabetes, thyroid diseases, etc. (4) History of psychoactive substance abuse or taking any medication that may affect cognitive function (e.g., glucocorticoids, beta-blockers, opioid analgesics, and central stimulants); and (5) Received ECT within 3 months.

Control group: matched with the depression group according to age and gender. Inclusion criteria: (1) age 18-60 years old; (2) junior high school education or above, able to cooperate with the completion of cognitive function tests and clinical assessment; (3) baseline status HAMD-17 total score <7 at the time of enrollment; (4) consent of the patients and their families, and to sign an informed consent form. Exclusion criteria: (1) pregnant or lactating women; (2) severe physical illnesses, such as severe liver and kidney disease, heart disease, hypertension, diabetes mellitus, thyroid disease, etc.; (3) history of abuse of psychoactive substances or use of any medication that may affect cognitive function (e.g., glucocorticosteroids, β-blockers, opioid analgesics, and central stimulants).

### Procedure and stimuli

**Figure 1:**
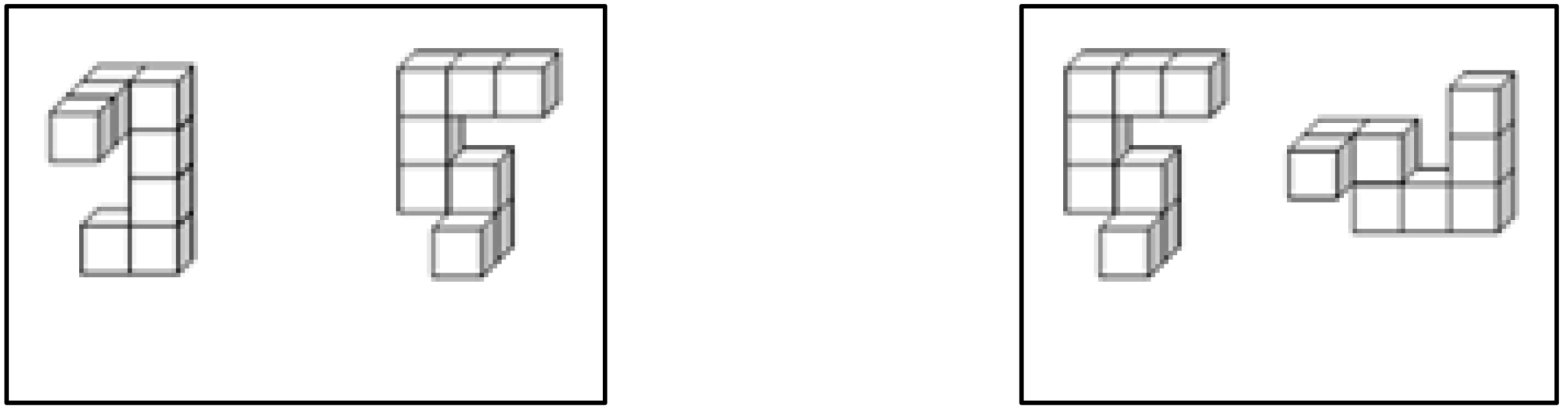
Stimuli example. On the left panel, the shapes are different that cannot be matched either by rotating or by mirroring them. For instance. On the right panel, the shapes are identical, and alignment is possible by 90 degrees yawing (rotating the second shape along the z-axis by 90 degrees) and then mirroring.

In the mental rotation task, participants were presented with two 3D images of abstract shapes consisting of 10 gray squares on a black background, as shown in the **Figure 1**. They were required to state whether the shapes in the two images were the same. For the pairs with identical shapes, the second shape can be aligned with the first image through rotation (around the X-axis or Y-axis, with a rotation range of 30 to 150 degrees,with 30-degree steps; or around the Z-axis by 90 degrees) or through mirroring. To accomplish the task, the participants needed to mentally rotate the images to check whether they matched. In each trial, the participants had to record their level of confidence in their responses on a 4-point scale, with 4 denoting “totally confident” and 1 indicating “not confident at all.” We had 36 pairs of identical shapes and another 36 pairs of different shapes in a randomized order, comprising a total of 72 trials. The figures below present some examples of the 3D images.

### Analysis

In this study, we measured average confidence as the metacognitive bias in each test. Within the test, the outcome of a trial in which the participant reports high confidence with a correct response is considered a metacognitive hit(Hit), while a trial wherein the participant provides a correct response while reporting low confidence is considered a metacognitive miss(Miss). A trial in which the participant reports high confidence while giving an incorrect response is considered a metacognitive false alarm(False Alarm), whereas low confidence reports corresponding to incorrect responses are considered to be correct metacognitive rejections(Correct Rejection).

We calculated the average confidence level of all participants to represent metacognitive bias. The Hit Rate(Hit Rate=Hit/(Hit+Miss)) and False Alarm Rate(False Alarm Rate=False Alarm/(False Alarm+Correct Rejection)) were calculated according to signal detection theory (3). The first-order task performance d’ was obtained by calculating the Z-scores(d′=Z(Hit Rate)−Z(False Alarm Rate)) of the standard normal distribution, which was used to measure the performance of participants in the test (the measure of performance in this task). Subsequently, the metacognitive sensitivity Meta-d’ was derived using the hierarchical Bayesian method (9) (Meta-d’ was calculated using the H Meta-d toolbox and implemented in MATLAB 2016a (MathWorks, Inc., Natick, MA, USA)). In addition, we quantified metacognitive efficiency (M Ration) with meta-d′/d′. Meta-d’/d’ is an indicator of metacognitive efficiency that evaluates how metacognitively efficient an individual is at a specific level of task performance. If meta-d’= d’, the observer is metacognitively “ideal.”

We did not analyze reaction time data, as we could not ensure that the hardware was identical across different experimental environment. Moreover, performance in the mental rotation task was not the focus of this study. We were interested in the closeness between confidence ratings and accuracy (metacognitive monitoring).

We aimed to compare the differences in metacognitive performance between a group with MDD and a healthy group using a mental rotation task; we conducted independent-samples t-tests using SPSS Statistics 25.0 to assess indicators related to their task performance and metacognitive abilities. These parameters included the accuracy of the first-order task performance (d’), metacognitive bias (average confidence), metacognitive sensitivity (meta-d’), and metacognitive efficiency (the M ratio). To provide more precise results with unequal sample sizes and variances, we used Welch’s t-test. The Pearson correlation coefficient was used to assess the correlation between the HAMD-17 scores and the metacognitive-related indices in individuals with MDD, as well as the relationship between metacognitive bias(average confidence)and d’, Meta-d’, and M Ratio.

## Results

**Figure 2:**
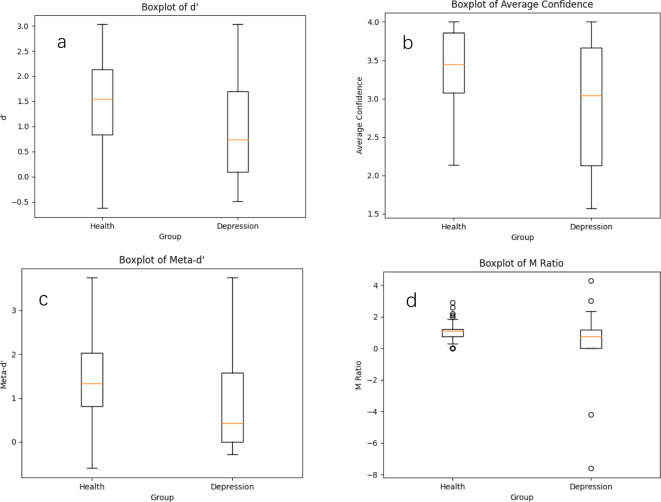
Boxplots of metacognitive parameters for both groups. Whiskers are defined to cover 90% of the data. Center lines show the medians, box limits indicate the 25th and 75th percentiles as determined by R software, whiskers extend to 5th and 95th percentiles, and outliers are represented by dots. **(a)** the difference in d’ between the healthy controls and the group with MDD. **(b)** the difference in average confidence between the healthy controls and the group with MDD. **(c)** the meta-d’ difference between the healthy controls and the group with MDD. **(d)** the M ratio difference between the healthy controls and the group with MDD.

We noted a statistically significant difference between the group with MDD and the healthy controls The d’ value in the signal detection theory exhibited a significant difference (t = -4.274, p < 0.001), which indicated that the healthy controls performed better in processing complex spatial information. For average confidence, the group with MDD scored significantly lower than the healthy controls (t = 4.280, p < 0.001), indicating that the healthy controls were more confident in their judgments when performing the mental rotation task. In terms of the meta-d’ value, the group with MDD scored significantly lower than the healthy controls (t = -3.540, p < 0.001), suggesting that the healthy controls had better metacognitive sensitivity and were more accurate in self-assessment. Regarding the M ratio, the group with MDD performed significantly worse than the healthy controls (t = -2.104, p = 0.039), implying that the healthy controls had better metacognitive efficiency. That is, they could utilize cognitive judgmental signals for self-assessment more effectively. **Figures 2a-d** outline detailed differences in performance. In sum, the healthy controls performed significantly better than the group with MDD on the mental rotation task as well as on the metacognitive parameters.

**Table 2:** Differences in task performance and metacognition on the mental rotation task between the group with MDD and the healthy controls.

Parameter	*t*	df	*p*	95% CI
d’	-4.274	127	<0.001	[-0.890, -0.327]
Average confidence	4.28	97	<0.001	[0.248, 0.668]
Meta-d’	-3.54	139	<0.001	[-0.889, -0.252]
M ratio	-2.104	78	0.039	[-0.757, -0.021]

According to **Table 3**, the HAMD-17 scores were significantly negatively correlated with the patients’, d’ value, average confidence, meta-d’ value, and M ratio, and the differences were statistically significant.

**Table 3:** Correlation between HAMD-17 scores and metacognition-related indicators in patients with MDD.

Indicators	d’	Average confidence	Meta-d’	M ratio
r	-0.468**	-0.601**	-0.457**	-0.362**

* indicates p<0.05; ** indicates p<0.01.

According to **Table 4**, the average confidence of patients was significantly positively correlated with d’,meta-d’,and M ratio, with statistically significant differences.

**Table 4:** Correlation between average confidence and d’, meta-d’, M ratio in patients with MDD.

Indicators	d’	Meta-d’	M ratio
r	0.552**	0.738**	0.273*

* indicates p<0.05; ** indicates p<0.01.

## Discussion

In this study, we observed that the MDD group exhibited significantly lower d-prime, average confidence, meta-d’, and M ratio compared to healthy controls on the mental rotation task, as shown in **Table 2**. Moreover, correlation analysis revealed that the severity of depression was significantly negatively correlated with metacognitive performance. Notably, we found a significant correlation between impaired metacognitive abilities and negative metacognitive bias(average confidence).

Cognitive decline is common in patients with MDD. Cognitive impairment is a key clinical manifestation of depression, which is characterized by a general decline in various domains, including psychomotor speed, attention, learning and visual memory, and executive functions. These cognitive symptoms may occur prior to other symptoms of depression and are important diagnostic criteria for depression, severely affecting daily life and work (10). The mental rotation task is a complex cognitive process that involves various cognitive functions such as spatial intelligence, attention, working memory, visual representational manipulation, motor planning, response control, information processing speed, and the activation and simulation of hand movements (11, 12, 17). Previous studies have demonstrated that mental rotation plays an important role in scene navigation (14), teleoperation (15) and the development of children’s logical arithmetic ability (16). In our study, we found that, on the mental rotation task, the first order task performance (d-prime) of the MDD group were significantly lower than those of the healthy controls, and it were significantly negatively correlated with the HAMD-17 scores, cognitive functions were more severely impacted by higher levels of depression. This indicates that the more severe the depression, the greater the impairment in cognitive function. A previous study measured the mental rotation task in 30 depressed patients and 28 healthy subjects, and the results showed that the mental rotation ability of depressed patients was impaired, and it was concluded that the impaired mental rotation ability could be used as a clinical auxiliary index for the diagnosis of depression (7). In 2014, Jiu Chen and colleagues (18) tested the performance of MDD patients and healthy controls using a mental rotation task, recording indicators such as reaction time and accuracy. They also employed ERP (Event-Related Potential) technology to conduct a temporal analysis of the electrophysiological mechanisms underlying mental rotation information processing in MDD patients, in order to reveal the cognitive model of visual-spatial information processing. The results indicated that the mental rotation ability of MDD patients is impaired. The ERP results showed that this impairment was particularly evident in the hand-related task, which may be associated with psychomotor retardation or slowness. Therefore, the mental rotation task not only effectively reflects the cognitive function status of patients with depression but also has the potential to become an important supplementary indicator for assessing the severity of depression.

In terms of metacognitive ability, this study has revealed significant differences in metacognitive bias, metacognitive sensitivity, and metacognitive efficiency among individuals with Major Depressive Disorder (MDD). First, we found that the negative metacognitive bias in the MDD group is characterized by a tendency to report low levels of confidence regardless of their actual task performance, and this bias is significantly correlated with the severity of depressive symptoms. This negative bias reflects an excessive caution or lack of confidence in self-assessment among individuals with depression. Previous clinical studies have shown that depression is associated with negative metacognitive bias. Barbara et al. (19) studied 30 patients with MDD and 30 healthy controls to examine their judgment and confidence. Although Barbara et al. found no differences in the metacognitive judgments of performance between the two groups of participants, the patients with MDD were significantly less confident in their judgment of themselves. Culot (20) conducted a study among healthy participants in which he used a combination of movies, pictures, and memories to induce negative and neutral moods in a metacognitive task. Participants reported lower levels of self-confidence in the negative condition than in the neutral condition. As found in most studies, most patients with MDD display low confidence or negative metacognitive bias (13, 21, 22). In other words, the negative tendencies of people with depression can cause them to be less confident or to develop a negative bias compared with people who are not depressed. A cross-diagnostic study indicated that patients with depressive/anxiety symptoms lacked confidence in their performance, and that another symptom of dimension—obsessive and intrusive thinking—was associated with increased confidence. Cognitive behavioral therapy (CBT) is able to significantly alleviate the symptoms of patients with depressive/anxiety disorders, accompanied by a significant increase in metacognitive confidence (23). Similar to CBT, the metacognitive bias of a group using antidepressants also increased. In sum, metacognitive bias is not a stable, fixed trait but fluctuates according to treatment responses, such as one’s state of anxiety or depression.

At the same time, we also found that individuals with Major Depressive Disorder (MDD) had significantly lower metacognitive sensitivity (Meta-d’) and metacognitive efficiency (M Ratio) compared to healthy controls, and was significantly correlated with negative metacognitive bias(average confidence). Metacognitive sensitivity reflects an individual’s ability to accurately assess their own task performance, that is, the ability to report high confidence when making correct judgments and low confidence when making incorrect judgments. Lower metacognitive sensitivity indicates that individuals with MDD have difficulty accurately distinguishing their successes from failures in self-assessment, which may be related to their general lack of confidence, that is, negative cognitive bias. Furthermore, metacognitive efficiency (M Ratio) is the ratio of metacognitive sensitivity to task performance, reflecting an individual’s ability to use cognitive signals to make self-assessments in a specific task. Lower metacognitive efficiency indicates that individuals with MDD have difficulty converting cognitive performance into accurate self-assessment, which may be related to their impaired cognitive function and overemphasis on negative information. It is worth noting that the relationship between metacognitive bias, sensitivity, and efficiency is not entirely independent. Negative metacognitive bias may lead patients to be overly cautious in self-assessment, thereby reducing metacognitive sensitivity and efficiency. Conversely, lower metacognitive sensitivity and efficiency may further exacerbate patients’ lack of confidence, creating a vicious cycle. This interaction suggests that when understanding the metacognitive impairments of individuals with depression, we need to consider the complex relationships between bias, sensitivity, and efficiency in a comprehensive manner.

This finding suggests that MDD patients are less efficient than healthy controls in their ability to process complex spatial information and assess their perceptual judgment performance. The traditional view is that metacognition is a relatively stable ability that does not change easily (24). However, a large number of recent studies support the idea that metacognition is modifiable or trainable and that prefrontal transcranial magnetic stimulation can alter metacognitive abilities (25). The integrity of metacognitive function is influenced by a unique neural network across the medial and lateral prefrontal regions (26). Moreover, fluctuations in patient alertness, stimulus sensitivity, or task difficulty can affect metacognition (27). For example, participants show lower metacognition in high-difficulty tasks than in low-difficulty tasks (28). In conclusion, when examining the effects of feedback on metacognition, the control of task performance is crucial, as confidence ratings are directly related to task performance.

Numerous studies have shown that the individual’s metacognition is susceptible to the feedback they receive in everyday life. Most feedback that patients with MDD receive is negative, which elicits unpleasant experiences and can impede cognitive functions (29). The symptom levels of depressed individuals are significantly and negatively correlated with their levels of self-confidence. In addition, they exhibit relatively low metacognitive sensitivity and efficiency, which implies that their abilities of self-regulation and self-control in cognitive processes are negatively impacted (5). Let us go one step further to hypothesize that the reason for reduced metacognitive efficiency in MDD patients may be related to negative metacognitive bias or low confidence levels. Besides, negative bias varies with symptom severity (30). A large number of empirical and theoretical studies suggest that the primary cause of the development and maintenance of depression is negative bias, which involves the unconscious, prioritized processing of negative information and can occur in different cognitive domains such as attention, interpretation, and memory. Wells et al. (31) argued that a problematic pattern of the self-regulatory executive function model in people with mood disorders comes in the form of rumination or active anxiety, which entails repetitive negative thinking as well as unhelpful cognitive and behavioral strategies. Once activated, negative thoughts expand and lead to persistent negative emotions. Chronic negative thinking and unhelpful cognitive strategies may diminish an individual’s self-efficacy (i.e., confidence in their ability to perform a given task). Reduced self-efficacy may lead to a lack of motivation and incentive for the individual to face challenges. Additionally, a lack of confidence may decrease motivation, which in turn affects one’s engagement in learning or performing tasks. A lack of engagement may generate a lack of investment in cognitive resources, thus reducing cognitive efficiency and affecting one’s decision-making abilities as a lack of confidence may cause indecision, risk avoidance, or the selection of suboptimal strategies (32). Over time, a vicious cycle develops wherein reduced confidence exacerbates symptoms of anxiety that are bound to affect attention, memory, and other cognitive processes, leading to a decline in cognitive functions. Another study confirmed a positive correlation between metacognitive efficiency and metacognitive bias (33), suggesting that if the same person were to increase their confidence as a result of any feature of an instruction or task, there may be an increase in their metacognitive efficiency, This is consistent with our research findings.

Our study has several limitations that necessitate further research. First, we did not account for the impact of therapeutic medications on MDD patients in our study. Some research has shown that antidepressant medications, such as selective serotonin reuptake inhibitors (SSRIs), can have a positive effect on patients’ cognition. Perhaps in the future, we could conduct more precise analyses based on a larger sample size. Second, our study did not include the state and metacognitive performance of MDD patients during remission periods. Future research could conduct longitudinal studies on case groups, combining pharmacotherapy and follow-up visits to better understand these aspects. Third, due to limited experimental conditions, we were unable to standardize the hardware equipment, which prevented us from conducting effective time-based data analysis. This limitation may have had an impact on the results of our study.

## Conclusions

We compared the metacognitive performance of MDD patients and healthy controls in a mental rotation task. The results showed that the metacognitive abilities of MDD patients were significantly impaired. Specifically, MDD patients exhibited significantly lower performance in first-order task performance, metacognitive bias, metacognitive sensitivity, and metacognitive efficiency compared to healthy controls. Additionally, the degree of metacognitive impairment in MDD patients was positively correlated with the severity of clinical symptoms. Notably, the impairment of metacognitive abilities was also significantly associated with negative metacognitive bias.

## Data Availability

The original contributions presented in the study are included in the article/supplementary material. Further inquiries can be directed to the corresponding author.
